# Supplementary material for: Biomass Nanoporous Carbon-Supported Pd Catalysts for Partial Hydrogenation of Biodiesel: Effects of Surface Chemistry on Pd Particle Size and Catalytic Performance
Source: Nanomaterials (Basel). 2021 May 28;11(6):1431. doi: 10.3390/nano11061431 (PMC8227727; doi:10.3390/nano11061431)
Supplement: Supplementary file 1 [file nanomaterials-11-01431-s001.zip › nanomaterials-1180632-supplementary.pdf]

nanomaterials

MDPI

Supplementary Materials: Biomass Nanoporous Carbon-Supported Pd Catalysts for Partial Hydrogenation of Biodiesel: Effects of Surface Chemistry on Pd Particle Size and Catalytic Performance

Parncheewa Udomsap [1], Sirasit Meesiri [2], Nuwong Chollacoop [3] and Apiluck Eiad-Ua [1,*]

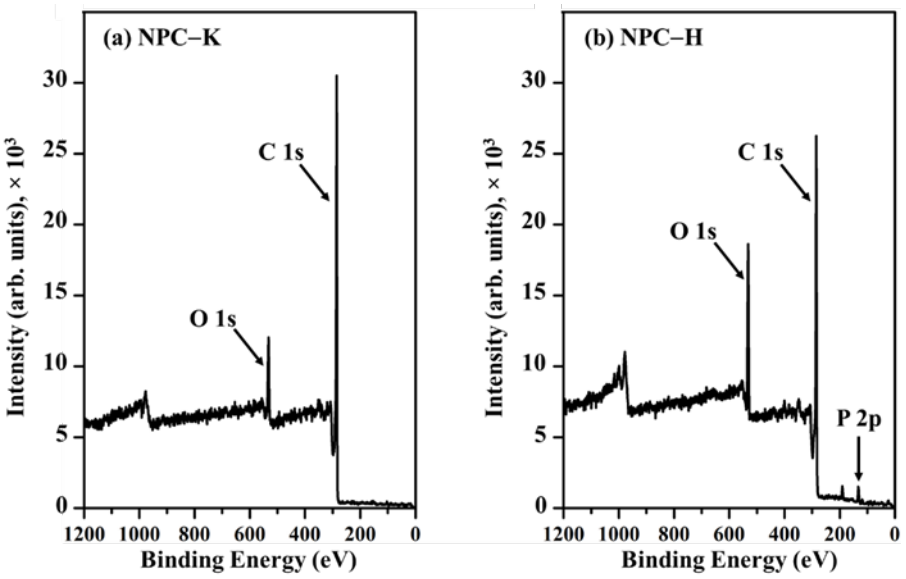

Figure S1. Wide scan XPS spectra of (**a**) NPC-K and (**b**) NPC-H.

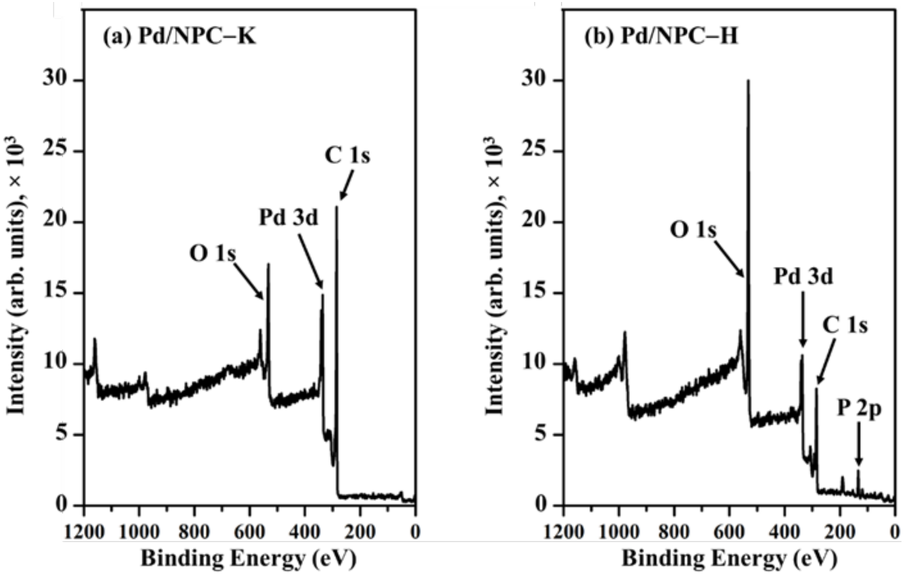

Figure S2. Wide scan XPS spectra of (**a**) Pd/NPC-K and (**b**) Pd/NPC-H.
